# Scalable, Color‐Matched, Flexible Plasmonic Film for Visible–Infrared Compatible Camouflage

**DOI:** 10.1002/advs.202303452

**Published:** 2023-10-27

**Authors:** Yuqin Xiong, Yitong Zhou, Junlong Tian, Wanlin Wang, Wang Zhang, Di Zhang

**Affiliations:** ^1^ State Key Laboratory of Metal Matrix Composite Shanghai Jiao Tong University Shanghai 200240 China; ^2^ Department of Electronic Science and Technology College of Big Data and Information Engineering Guizhou University Guiyang 550025 China; ^3^ College of Electronics and Information Engineering Shenzhen University Shenzhen 518060 China

**Keywords:** anodic oxide alumina, background matching, camouflage patterns, multiband infrared camouflage technology

## Abstract

The multispectral compatible infrared camouflage technology is implemented these days to counter the developing infrared detectors and detectors of other bands. However, the conflict between delicate optical structures and scalable procedures has significantly impeded the development and application of multispectral‐compatible camouflage technology. Therefore, a semi‐open Fabry‐Perot structure is introduced, and the color and infrared emissivity by structural parameters for color‐matched visible‐infrared compatible camouflage are modulated. The prepared compatible camouflage film exhibits visible camouflage by the minimum color difference of 1.6 L*a*b* (under desert background) and infrared camouflage by low emission (ε_3–5 µm_ ≈ 0.17 and ε_8–14 µm_ ≈ 0.143). Due to its flexibility and scalability, the compatible camouflage film can be applied in practical applications and exhibits desirable visible and infrared camouflage performance in different battlefield backgrounds.

## Graphical abstract

A simple method is used to prepare semi‐open Fabry‐Perot structured materials and achieve background matching by modulating the color and infrared emissivity of with structural parameters. The prepared compatible camouflage film has a small visible color difference and low infrared emissivity. With its flexibility and scalability, the compatible camouflage film can be used for different shapes of targets and different backgrounds.

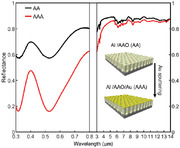

## 1 Introduction

Camouflage, also called cryptic coloration, is the ability of certain animals to merge with their surroundings to protect against predators or to attack prey.^[^ ^1^ ^]^ In nature, certain animals can change their own color and texture according to the surrounding environment to merge with the background.^[^ ^2^, ^3^ ^]^ Similarly, in the human world, camouflage is required to counter the developing detector technology in military and surveillance applications. Therefore, it is necessary to reduce the detectability of a target at different detectable wavelengths by adjusting its characteristic signal to be the same or comparable to the background environment.^[^ ^4^, ^5^, ^6^ ^]^ With the popularity of infrared detection and surveillance systems, the visible and mid‐infrared ranges are the two most prevalent and important wavebands in camouflage technology.^[^ ^7^, ^8^, ^9^ ^]^ To achieve spectra modulation in both visible and infrared ranges, materials with nanostructures have been explored and promising research progress has been accomplished.^[^ ^5^, ^10^, ^11^, ^12^, ^13^ ^]^ By modifying the structural parameters of the material surface, researchers achieve optical modulation at different wavelengths by changing the optical properties (reflection, absorption, and transmission) of the intrinsic material via interference and scattering.^[^ ^14^, ^15^, ^16^, ^17^, ^18^ ^]^ At the same time, nanostructured materials have some distinct advantages over conventional chemically dyed camouflage materials, such as good thermal and chemical stability and long durability, and are therefore expected to be at the forefront of applied research.^[^ ^19^ ^]^

According to recent visible‐infrared compatible camouflage studies, various nanostructured configurations, such as multilayers,^[^ ^20^, ^21^ ^]^ photonic crystals,^[^ ^22^, ^23^ ^]^ plasmonic,^[^ ^15^ ^]^ and metamaterials,^[^ ^5^, ^24^, ^25^, ^26^, ^27^ ^]^ have produced different interference colors in the visible range, while exhibiting ultra‐low emissivity in the infrared range. The majority of nanostructure‐based multiband camouflage studies are currently at the fundamental research stage due to their limited size and complicated fabrication procedure. To get closer to the application scenario, experimental attempts at real applications need to be done, and thus it is necessary to address two issues: background matching and application feasibility.

In visible camouflage, the best‐known strategy is background matching^[^ ^1^ ^]^: by matching the color, brightness, and pattern of the material to the background, to minimize the detection probability. Real‐world battlefield terrain is complex and diverse, requiring different visible camouflage clothing or painting to merge into the background. Recent research has also focused on camouflage material matching with the background, not only for the visible region but also for the infrared region.^[^ ^28^, ^29^ ^]^ Among these infrared background matching strategies, the infrared signature patterning is more suitable for camouflage of low‐temperature objects because infrared patterns will show high infrared signatures on high‐temperature objects as well. On the other hand, the signature lowering strategy has a wider range of applicability and is more suitable for hiding high‐temperature objects in backgrounds without infrared feature differences. Overall, visible‐infrared compatible materials need to match the visible color of the background while having very low infrared emissivity. In terms of application feasibility, considering the material‐target combination, the scalable fabrication and flexibility of the camouflage material (in the case of bulk material) are both essential. Metamaterials inevitably require nanopatterning, which implies complex fabrication processes (including electron beam lithography) that not only hinder scalability but are also expensive.^[^ ^4^, ^30^ ^]^ For multilayer materials with simple fabrication processes, the samples are often too thick to be applied to complex surfaces.^[^ ^20^, ^22^ ^]^ Therefore, an efficient approach that enables background matching and application feasibility in the field of visible infrared compatible camouflage is urgently required.

To address the above challenges, we proposed a multispectral camouflage approach based on a semi‐open Fabry–Perot (F–P) nanocavity structure which utilizes surface plasmonic absorption and thin film interference to generate color for visible camouflage and low infrared emissivity of the substrate for infrared camouflage. Compared with the conventional F–P cavity, we choose electrochemically fabricated porous anodic aluminum oxide (AAO) as the dielectric layer in the F–P cavity, which results in the patterning of the surface metal and achieves a fuller range of colors and larger scale. Taking advantage of the AAO, we can effectively achieve color‐changing ability by modifying the structural parameters through the fabrication process. Thus, we adjusted the experimental parameters to color‐match the camouflage flexible film with two typical battlefield backgrounds: woodland and desert. To address the practical possibilities of the proposed camouflage material, we performed camouflage performance tests on the camouflage color pattern and evaluated its ability to be applied to real models.

## 2 Results and Discussion

### 2.1 Concept of Multiband Camouflage and Structure Design

For visible infrared compatible camouflage technology, the background matching strategy is manifested as color matching in visible band camouflage and radiation temperature matching in infrared band camouflage. Thus, the requirement of materials is different for different wavelengths: in the visible (VIS, 380–780 nm) range, the material is required to have color controllability; in the infrared (IR) atmospheric window, mid‐wave infrared (MWIR, 3–5 µm) and long‐wave infrared (LWIR, 8–14 µm) ranges, the material is required to possess low emissivity. To avoid detection by telescopes and surveillance systems, the optical properties of the material surface should be regulated to make it compatible with the surrounding environment (e.g., woodlands and deserts). Furthermore, considering the detection system in the mid‐infrared range, the thermal signal of the hot object should be suppressed in order to reduce the detection probability of the infrared camera. The desired visible infrared compatible camouflage system is illustrated in Figure S1 (Supporting Information).

**Figure 1:**
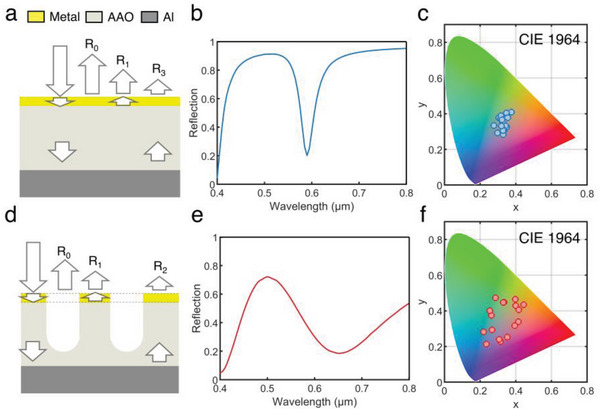
Mechanism and structure illustration. a,d) Schematic diagrams of light propagation in two types of structures: typical F–P cavity and semi‐open F–P cavity. Simulated reflectance spectrum (b,e) and calculated color coordinates (c,f) for the two structures.

Generally, the most typical type of thin film structure is the F–P nanocavity based on metal‐dielectric‐metal (MDM) configuration (shown in **Figure** **1**a). In order to reduce the thermal signal of the surface, the highly reflective film used for the bottom layer is an aluminum foil, which has an ultra‐low infrared emissivity (≈0.01) and is very flexible as a substrate. Meanwhile, the material of the middle dielectric film is alumina, which is almost transparent in the mid‐infrared region, to avoid the effect on infrared camouflage.^[^ ^6^ ^]^ When the top metal layer is Ag, the reflection simulation result suggests that narrowband absorbers appear at the wavelength of the reflective valleys and show maximum peak‐to‐valley fluctuation (Figure 1b).^[^ ^31^ ^]^ Apart from the sharp absorption peaks, the reflectivity stays close to 1 in the rest of the wavelength, which results in little perceptible color in the typical F–P nanocavity structure (Figure 1c). The narrowband spectral response results from the multiple‐beam interference that happened on the surface of the highly reflective top metal layer (Note S1, Supporting Information).

In order to generate more apparent color, the wide and flat reflectivity peak of the F–P nanocavity structure needs to be narrower to increase the saturation of color. It is also needed to have high absorption through a wide range of wavelengths to reduce the lightness of the color. To achieve that, we use a semi‐open F–P nanocavity structure by changing the middle dielectric film from flat alumina to AAO, which means that the top flat metal film has been replaced by a porous metal film (Figure 1d). The porous metal film introduces more light into the dielectric film, which increases the light interference in all phase changes (Note S1, Supporting Information). The relocation of metal distribution increases the plasmonic resonation between isolation metal clusters and enhances the absorption energy when destructive interference occurs in the top metal layer.^[^ ^9^, ^32^ ^]^ The simulated reflection spectra show appropriate peaks and valleys when constructive interference and destructive interference occur (Figure 1e).^[^ ^33^ ^]^ Due to the wavelength‐selective reflection from the surface, a distinct structural color range is expected to occur as the thickness of the AAO layer varies (Figure 1f). By adjusting the type of surface metal layer, thickness, unit cell size, and pore diameter of AAO, etc., the range of structural colors can be further expanded and appropriate materials and structures can be selected according to the strategy of visible camouflage color matching.

### 2.2 Materials and Structural Dependencies

**Figure 2:**
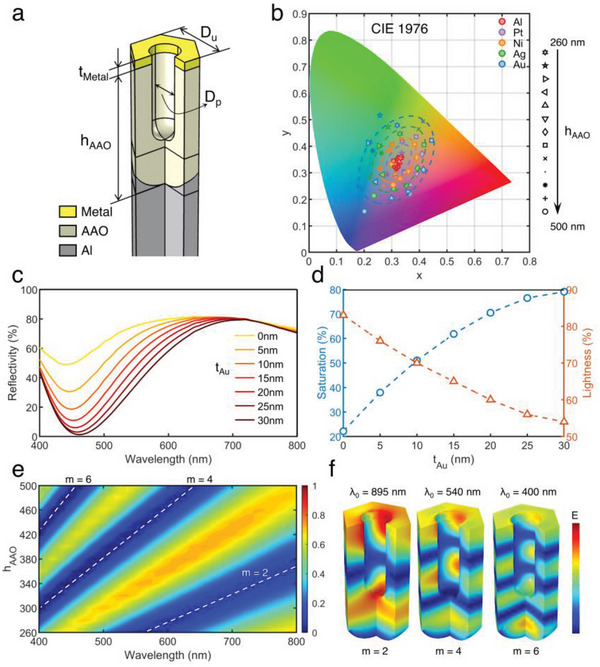
Material and structure dependency. a) Concept illustration of the semi‐open F–P structure for optical simulation. b) Color gamut of diverse materials for the top layer. c) Visible reflectance spectra of simulated results depending on the Au thickness. d) Color saturation and lightness of simulated results depending on the Au thickness. e) Visible reflectance spectra of simulated results depending on the AAO thickness. f) Local electric field distribution in the model with the same thickness (h_AAO_ = 420 nm) of a dielectric layer under different incident wavelengths.

To further investigate the material and structural dependence of the aforementioned semi‐open F–P nanocavity structure, we performed optical simulations in the VIS and IR regions. **Figure** **2**a depicts one of the cells used for the optical simulation of the semi‐open F–P nanocavity structure, composed of a top metal layer, a middle AAO layer, and a bottom Al layer (Metal‐AAO‐Al, MAA), with the following structural parameters set: top metal thickness *t*_Metal_, AAO cavity length *L*_AAO_, unit cell size *D*_u_, and pore diameter *D*_p_. Figure 2b shows the color representation on the Commission Internationale de l'Eclairage (CIE) color space depending on various top metal materials and the *L*_AAO_ varying from 260 to 500 nm. The simulation results showed the broadest range of colors on the CIE 1964 diagram when the top layer metal is Au, indicating that this material configuration (Au‐AAO‐Al, AAA) presents higher color saturation and broader modulation (Figure S3, Supporting Information).

To investigate the role of top Au layer played in this structure, the reflectance spectra of the semi‐open F–P nanocavity structure with different Au thicknesses were also simulated. The simulation results showed that the reflectance decreased at a certain wavelength with the increase of the top Au layer thickness (Figure 2c). An energy loss analysis on the simulation results was performed to obtain the total power dissipation density distribution of the model with and without the Au layer in order to investigate the effect of the Au layer component on increasing the absorption efficiency of the model for a specific wavelength light incidence (Figure S4a, Supporting Information). The analysis results showed that the majority of the absorption loss happened in the Au layer, thus, the overall absorbed power of the model has been improved and the overall reflectivity has been reduced in the presence of the Au layer. The enhanced absorption at a certain wavelength not only reduced the lightness of the corresponding color but increased its saturation. When the thickness of the top Au layer increases from 0 to 30 nm, the color saturation increases from 30.4% to 80% while the lightness decreases from 83% to 54% (Figure 2d). From the simulation results, as the Au layer becomes thicker, the absorption of the model at specific wavelengths increases, resulting in a smaller FWHM (Full Width at Half Maximum). The high reflectance in a narrower wavelength band resulted in calculated colors with better monochromaticity and higher saturation.

Apart from lightness and saturation, the third element of the LSH color system, hue, is the key factor in achieving color modulation ability and multiple background camouflage, which is determined by the reflection peak position of the reflection spectrum. As mentioned above, the thickness of the top Au layer does not affect the peak position of the reflection spectrum, so we simplify the model into a three‐layer thin film interference structure (Note in Supporting Information). The peaks and valley positions depend on the wavelength of interferences on the surface of the model, determined by the thickness of the dielectric layer of the semi‐open F–P cavity, which is the thickness of the anodic aluminum oxide layer (h_AAO_) in this model. By studying the simulation results of the models with different h_AAO_, we found that the positions of the reflection peaks and valleys of the models support the results of the theoretical calculations well. The refractive index of alumina is greater than that of air and aluminum. When the interference order m is even, constructive interference occurs and the reflection spectrum has a minimal value; when the interference order m is odd, destructive interference occurs and the reflection spectrum has a maximal value (Note S1, Supporting Information). The dashed lines in Figure 2e indicate the positions of the reflection valleys for different models when m is equal to 2, 4, and 6. When *h*_AAO_ is equal to 420 nm, the model has the reflection valleys of these three interference orders at 895, 540, and 400 nm, and the electric field distribution of the model at these three wavelengths is shown in Figure 2f. The constructive interference was matched with the plasmonic layer, and the initial constructive interference combined with the absorption of the gold layer further reduced the intensity of reflected light. In the same model, the phase difference caused by the optical path difference caused by different wavelengths of light is different, resulting in different orders of interference. With the same interference order, such as *m* = 4, the phase of the optical path difference caused by different wavelengths of light passing through models of different heights is the same, i.e., 2π, so the electromagnetic field distribution in different models is similar (Figure S4b, Supporting Information). The thickness of the AAO layer is the key parameter to control the position of the reflection peak and further control the hue of color. The pore size of the AAO layer (*D*_p_) is another key parameter. At the same AAO thickness, the optical path difference caused by the same wavelength of light passing through the models with different pore sizes is different, so the electromagnetic field distribution in Figure S5a (Supporting Information) is different. The increased pore size affects the refraction coefficient of the intermediate AAO layer, which causes a blue shift of the reflection peaks by changing the wavelengths when interference occurs (Figure S5a, Supporting Information). Overall, compared to the thickness of the AAO layer, the pore size has a lesser effect on the reflection peak position of the model.

The structural parameters of the model not only affected the optical response in the visible region, but also affected its optical performance in the infrared region. Compared to AA structure, the emissivity of the AAA structure increased with the thickness of the surface Au layer, especially in the 3–5 µm range (Figure S6a, Supporting Information). The Au layer on the surface partially absorbed the infrared light and increased the emissivity of the structure, according to the total power dissipation density distributions of the AA and AAA structures in 3 µm (Figure S6b, Supporting Information). To prevent a large increase in IR emissivity, the thickness of the Au layer must be kept under control. Apart from the surface Au layer, Figure S7a (Supporting Information) showed the simulated reflectance spectra of the models with different AAO thicknesses in the IR region. When the AAO thickness (h_AAO_) was relatively thin (<250 nm), the model had high reflectance in the entire IR detection region. The increasing AAO thickness significantly raised the emissivity of the model in the MWIR region,^[^ ^34^ ^]^ with an insignificant effect in the LWIR region (Figure S7b, Supporting Information). The average IR emissivity of the simulation results is 0.077 (MWIR) and 0.018 (LWIR).

### 2.3 Characterization of the AAA Structured Material

**Figure 3:**
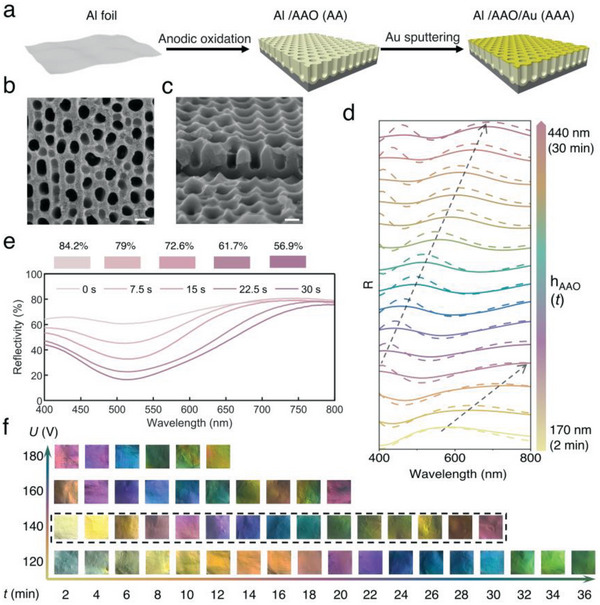
Characterizations in the visible range. a) Fabrication process of the AAA‐structured film. b) Top surface and c) cross‐sectional SEM images of the AAA‐structured film. The scale bars of the SEM images are 200 nm. d) Reflectance spectra of experimental and simulated results of AAA‐structured film depending on the AAO thickness. The dashed lines are used to trace the locations of reflection peaks with tuning. e) Reflectance spectra of experimental results depending on the sputtering time. f) Photographs of AAA‐structured films with different oxidation times and voltages.

Through 3D simulations, we determined the optical reflection spectrum of models with different structural parameters. To experimentally validate the optical behavior of the model, we fabricated the thin film material with a semi‐open F–P cavity structure (AAA material configuration). The fabrication process of the AAA thin film is shown in **Figure** **3**a. The process started with pre‐treated Al foil as substrate and a thin AAO dielectric layer was grown on the surface of the Al substrate by anodic oxidation. Next, Au particles were sputtering on the top of the AAO dielectric layer. Through scanning electron microscopy (SEM), we can determine the geometric information of the AAA thin film, and the typical top and cross‐sectional view of the film are shown in Figure 3b,c. By measuring the geometric parameters of all the samples fabricated in different conditions, we found the relationship between fabrication parameters and structure (Figures S8 and S9, Supporting Information). The thickness of the AAO layer (h_AAO_) increased as oxidation time (*t*) increased. The growth speed of the AAO layer was faster with a higher oxidation voltage(*U*). The higher oxidation voltage also resulted in a wider unit cell size (*D*_u_) of the quasi‐periodic AAO layer. According to cross‐sectional electron micrographs of the AAO layer, the pore sizes of AAO were all close to 100 nm and did not vary significantly at specific oxidation voltages (Figure S10, Supporting Information). With little fluctuation in the pore diameter of AAO, the refractive index of thicker AAO is closer to that of air ($n_{{\mathrm{Al}}_{2}O_{3}}>n_{\mathrm{air}}$) at the same oxidation voltage because the ratio of alumina to air at the top of AAO is smaller than that of alumina to air at the bottom. The refractive index test results indicated that the refractive index decreases with increasing AAO thickness at the same oxidation voltage (Figure S11, Supporting Information).

To study the optical characterization of the AAA thin film in the visible region, we obtained complete color series samples with different fabrication parameters, and the reflection spectra of all the samples were measured using a UV–vis–NIR spectrometer. Figure 3d shows the simulated reflectance spectra and the corresponding experimental reflectance spectra of samples with different AAO thicknesses under normal incidence (*U* = 140 V). As the thickness of AAO increases, the position of simulated reflection peaks gradually red‐shifts. And by comparing the simulated and experimental results, it can be easily observed that they are extremely similar in terms of the shape and tendency of the spectra. The reflectance peak intensities of the simulated and experimental spectra are slightly different due to the dimensional variation of the AAO layer during the fabrication. The positions of reflection peaks induced by interferences from experimental spectra are in good agreement with those from corresponding simulated spectra. The peak position shifted to a longer wavelength with the increase of oxidation time and completely covered the entire visible range.^[^ ^35^ ^]^ The structural parameters of the AAO layer are controlled by the anodic oxidation process, and the thickness of the top Au layer can be controlled by the sputtering time. Similar to the simulation results, the Au layer on the surface will produce strong plasmon resonance absorption at the wavelength of constructive interference occurring on the surface, causing a dip of the material reflectance spectrum at a specific wavelength with increasing Au layer thickness (Figure 3e). As the Au layer thickness increases, the FWHM of the spectrum gradually becomes smaller and the saturation of the material gradually increases. The significant plasmon resonance absorption resulted in a reduction in the material's overall brightness, and the brightness of the sample fell from 84.2% to 56.9% as the top Au layer's thickness increased from 0 to 12 nm. Due to the random nano‐size of the sputtered gold, thinner Au layers are sufficient to increase saturation and reduce brightness compared to the simulated results. The photos of the actual prepared films with different sputtering times (different Au layer thicknesses) are shown in Figure S12 (Supporting Information).

The pictures of all the fabricated films are shown in Figure 3f. The colors of the samples varied from yellow to green and were determined by the oxidation time (*t*) and oxidation voltage(*U*). After observing the colors of the series of samples at different *U*, we found that higher oxidation voltage resulted in a faster‐grown speed of *h*_AAO_ which means a swifter change of color hue. For comparison, the corresponding pictures of all the fabricated samples before Au sputtering (Figure S13, Supporting Information) have shown that the top Au layer plays a great role in the coloration of samples. The top Au layer significantly increases the saturation of the sample's color without changing the Hue. According to Figure S14 (Supporting Information), the reflectance of the sample in the visible range is low at all angles. Moreover, as the incident angle increases, the reflection peak of the sample is slightly blue‐shifted. Also, the comparison between Figure S14a and b (Supporting Information) shows that the sputtered gold layer has little influence on the angle dependence of the structure. The pictures taken at different viewing angles (0°–60°) of the thin film also confirmed that (Figure S15, Supporting Information).

**Figure 4:**
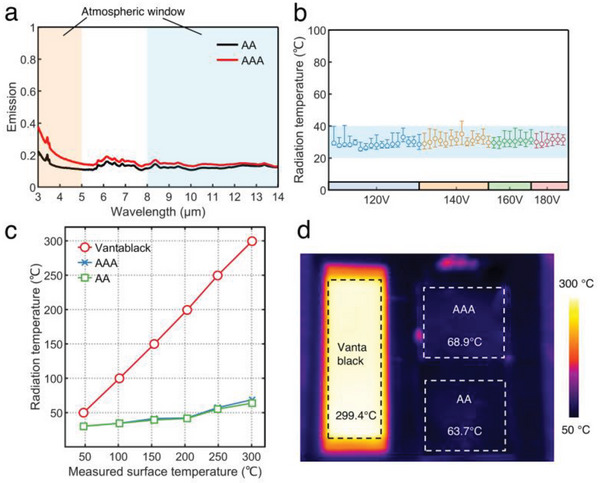
Characterizations in the infrared range. a) Emission spectra of the AA‐ and AAA‐structured films in the infrared range. b) Radiation temperatures at 100 °C surface temperature for all series of AAA‐structured films. c) Measured radiation temperatures for Vantablack, AA‐structured film, and AAA‐structured film under different surface temperatures. d) Thermal image for Vantablack, AA‐structured film, and AAA‐structured film at the highest surface temperature of 300 °C, and average radiation temperatures of each material are indicated in the dashed boxes.

As for the infrared region, we used Fourier transform infrared spectroscopy (FTIR) combined with int sphere to measure the infrared emission spectra of the samples (**Figure** **4**a). As shown in the emission spectra, the Au layer on the surface of the AAA‐structured film slightly increased its emissivity in the 3–5 µm region in comparison with that of the AA‐structured film. The measured band emissivity was 0.12 (3–5 µm) and 0.17 (8–14 µm) for the AA‐structured film compared to 0.124 (3–5 µm) and 0.143 (8–14 µm) for the AAA‐structured films. In addition, an infrared emissivity meter was used to measure all samples in Figure 3f and obtain an average infrared emissivity of 0.15 (80 °C).

Furthermore, thermal images of the samples were taken by a thermal camera (LMIR) to measure the radiation intensity and IR apparent temperature. The radiation temperatures of the samples with different voltage series were significantly reduced to between 20 and 40 °C, with an average radiation temperature of 30.1 °C, when the set actual temperature was 100 °C (Figure 4b). Under indoor configuration, by setting the surface temperature of the sample from 50 to 300 °C, all of the radiation energy of the sample was collected by the thermal imager, and the corresponding radiation temperature was obtained (Figure 4c). Compared to Vantablack (IR emissivity>99.7%), samples with AA and AAA structures have lower band emissivity, resulting in lower radiation temperatures and radiation intensities. The Au layer on the surface of the AAA‐structured sample slightly raised its radiation temperature (compared to the AA structure). When the measured temperature of the surface is 300 °C, the radiation temperatures of sample AAA (68.9 °C) and sample AA (63.7 °C) were 230.7 and 235.7 °C lower, respectively, than that of Vantablack (299.4 °C). In the spectral range of the thermal imaging camera, both the AA sample and AAA sample in the infrared image (Figure 4d) also showed lower radiation intensities than that of Vantablack, demonstrating better infrared camouflage performance. Furthermore, thermal images (Figure S16, Supporting Information) taken at different viewing angles of the film (0°−60°) demonstrated the angular independence of the film in the infrared region.

### 2.4 Visible–Infrared Compatible Camouflage Demonstration

**Figure 5:**
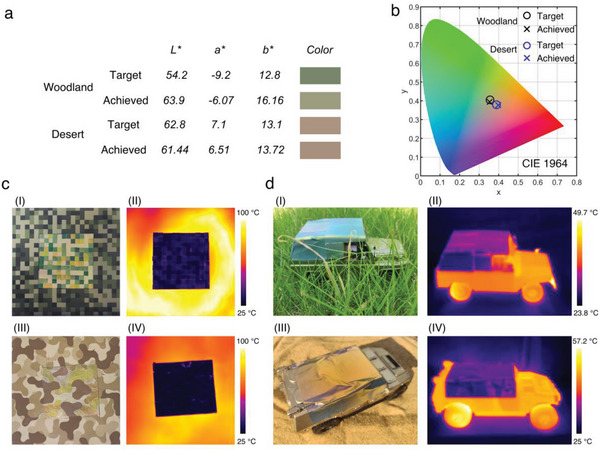
Color‐matching for visible and infrared camouflage. a) Calculated L*a*b* values and corresponding colors of camouflage targets and woodland and desert camouflage films. b) CIE coordinates of the target and achieved colors for woodland and desert camouflage situations. c) Visible (I) and infrared (II) images of digital woodland camouflage. Visible (III) and infrared (IV) images of disruptive desert camouflage patterns. d) Outdoor photograph of model car with woodland (I) and desert (III) camouflage film coating. Indoor thermal image of model car with a woodland (II) and desert (IV) camouflage film coating.

After demonstrating the color modulation ability and ultra‐low IR emissivity of the AAA structured material, we turned to explore its application to visible‐infrared compatible camouflage. Owing to the scalability and flexibility of the AAA structured material (Figure S17, Supporting Information), its application as a camouflage film is beneficial for coating and large‐scale production. AAO layer with a certain degree of flexibility is well adhered to the aluminum substrate with good flexibility. The AAO layer on the material's surface will bend when applied to the target object together with the aluminum substrate, maintaining the effectiveness of the visible infrared camouflage without peeling off. Micro‐region bending of AAO enables visualization of substantial bending at large scales. To verify the color‐matching ability of the camouflage film in a typical battlefield environment, we evaluated the film's color against typical camouflage colors and calculated the color differences (**Figure** **5**a). The calculated color of the woodland camouflage film had a similar hue to the target camouflage green color (#78866B), but was less bright than the target color. While the calculated color of the desert camouflage film is approximately the same as the target desert sand camouflage color (#AB9381), with a small color difference (Δ*E*) of only 1.6 L*a*b* (CIE 1976), which can be considered as there is no color difference in the bare human eye. The chromaticity diagram showed the target and achieved colors for woodland and desert on the CIE 1964 color space (Figure 5b). Whether woodland or desert camouflage, the chromatic coordinates of the target and achieved colors were well matched in the CIE 1964 color space.

Then, visible‐infrared camouflage was realized with the two types of camouflage material fabricated using digital woodland camouflage and disruptive desert camouflage patterns (Figure 5c). The two camouflage patterns both achieve effective visible camouflage as well as infrared camouflage by suppressing the high thermal signatures of the background in the infrared images. Figure 5d shows the optical and IR images of two alloy car models with woodland and desert camouflage film coating (0.08 × 0.08 m^2^) under sunlight and indoors. As shown in Figure 5d(I,III), the woodland and desert camouflage films can cover the initial surface color of the models, demonstrating similar colors to the background environment, and allowing the covered objects to blend in with the surroundings. Meanwhile, the areas coated by woodland and desert camouflage films show lower radiation temperatures compared with other areas on the models in Figure 5d(II,IV), appearing nearly as cool as the environment. This result demonstrates that the woodland and desert camouflage films could be practiced as multispectral camouflage coatings to conceal hot objects from both visual and IR detection. In other words, the woodland and desert camouflage films can realize background matching in both visible and infrared regions.

## 3 Conclusion

In this paper, we successfully demonstrated the potential application of semi‐open F–P cavity structure in the field of visible‐infrared camouflage. The fabricated visible‐infrared compatible camouflage films can be applied in background matching for different terrain backgrounds. We demonstrated the possibility of various color generation from semi‐open F–P cavity structures with AAA material configurations and investigated the relationship between The LSH color system and structural parameters. And through color‐matching strategies and modulation of fabrication parameters, the minimum color difference between the camouflage film and the target color was achieved at 1.6 L*a*b* in the case of desert camouflage. In terms of infrared camouflage technology, visible‐infrared compatible camouflage film exhibited low emissivity in the MWIR and LWIR wavelength ranges, less than 0.2 and 0.15, respectively. We quantitatively evaluated the infrared camouflage performance based on the reduction of thermal radiation temperature by the compatible camouflage film. The temperature difference between the thermal radiation temperature of the camouflage film and the actual temperature in the LWIR thermal image exceeded 230°C when the actual surface temperature was 300 °C. The compatible camouflage materials proposed in this work have strong potential for real‐world applications due to their excellent camouflage performance, flexibility, and large‐scale production. To further promote their practical applications, research on the reverse design of camouflage film color by actual battlefield background color is also required.

## 4 Experimental Section

### Fabrication and Material Characterization

The aluminum foil (purity: 99.999%) was rinsed three times with acetone, deionized water, and anhydrous ethanol to remove oil from the surface. Subsequently, one‐step anodic oxidation was carried out in a phosphoric acid solution of 0.2–0.4 mol L^−1^ at 5 °C with the platinum sheet electrode as the cathode and the platinum sheet electrode kept parallel to the aluminum foil with a constant voltage of 120–180 V for a range of time between 2 and 38 min. After anodization, the samples were washed twice with deionized water and anhydrous ethanol, respectively, and dried in an oven at 60 °C for at least 6 h. Finally, a thin Au layer was sputtered on the surface of the camouflage film by direct current ion sputtering technique with a sputtering time of 5–30 s. Spectroscopic ellipsometry (Semilab SE‐2000) was used to determine the gold layer's thickness on a silicon wafer that was simultaneously sputtered with gold. Surface morphology was characterized using an ultrahigh‐resolution scanning electron microscopy (RISE‐MAGNA). The refractive index of the interlayer was measured by spectroscopic ellipsometry (Semilab SE‐2000).

### Spectral Property Measurement

Optical properties in the visible–near‐infrared band (250‐2500 nm) were measured on the sample surface with an integrating sphere attachment of a UV–vis–NIR spectrophotometer (Lamda 950). The spectra with different incident angles were measured with a URA attachment of a UV–vis–NIR spectrophotometer. Since the bottom metal layer of samples is very thick and has a thickness that exceeds the skin depth of the incident light, the transmission is zero. And according to Kirchhoff's law of thermal radiation, the emissivity is equal to the absorptivity. Therefore, its emittance is considered ε(λ) = α(λ) = 1 – ρ(λ) – τ(λ), where ε, α, ρ, and τ describe the emittance, absorbance, reflectance, and transmittance, respectively. Optical properties in the mid‐ and far‐infrared band were tested on the sample surface using a Nicolet 6700 Fourier Transform Infrared Spectroscopy (FTIR) with an integrating sphere attachment. The test temperature was room temperature, and the wavelength range was 2.5–25 µm. IR‐2 infrared emissivity meter was used to measure the average infrared emissivity under 80 °C.

### Optical Images and Temperatures Measurement

To obtain infrared images, infrared thermal cameras (Fluke Ti60+ and Guide PS610) with a spectral response of 7.5–14 µm were used. In addition, a thermostatic heating table was used to maintain the temperature of the samples at the set temperature, 50–300 °C. The radiation temperature was measured from infrared thermal images. The surface temperature was measured by thermocouples (Omega 5TC‐TT‐K30‐36). The thermocouple was fixed to the upper surface of the samples.

### Simulations

The optical simulation was carried out using the radio frequency module (electromagnetic waves, frequency domain) in COMSOL Multiphysics 6.1.

## Conflict of Interest

The authors declare no conflict of interest.

## Author Contributions

W.Z., J.T., and D.Z. conceived and designed the research. Y.X. fabricated the samples and conducted experiments. Y.X. and W.W. designed the structure and performed the simulation. Y.X. and W.Z. analyzed the results and wrote the manuscript. Y.Z. helped with manuscript revisions and supplemental experiments. All authors approved the final version of the manuscript.

## Supporting information

Supporting Information

## Data Availability

The data that support the findings of this study are available from the corresponding author upon reasonable request.
