# Supplementary material for: Scalable, Color‐Matched, Flexible Plasmonic Film for Visible–Infrared Compatible Camouflage
Source: Adv Sci (Weinh). 2023 Oct 27;10(35):2303452. doi: 10.1002/advs.202303452 (PMC10724423; doi:10.1002/advs.202303452)
Supplement: Supplementary file 1 — Supporting Information [file ADVS-10-2303452-s001.pdf]

ADVANCED SCIENCE
Open Access

Supporting Information

for *Adv. Sci.*, DOI 10.1002/advs.202303452

Scalable, Color-Matched, Flexible Plasmonic Film for Visible–Infrared Compatible Camouflage

Yuqin Xiong, Yitong Zhou, Junlong Tian, Wanlin Wang, Wang Zhang* and Di Zhang*

Scalable, color-matched, flexible plasmonic film for visible infrared compatible camouflage

Yuqin Xiong[1], Yitong Zhou[1], Junlong Tian[2], Wanlin Wang[3], Wang Zhang[1] & Di Zhang[1]

Correspondence: Junlong Tian (jltian169@163.com) or Wang Zhang (wangzhang@sjtu.edu.cn)

[1]State Key Laboratory of Metal Matrix Composite, Shanghai Jiao Tong University, Shanghai 200240, China

[2]Department of Electronic Science and Technology, College of Big Data and Information Engineering, Guizhou University, Guiyang 550025, China

[3]College of Electronics and Information Engineering, Shenzhen University, Shenzhen 518060, China

Contents:

Supplementary Figures 1-17 and Supplementary Note 1.

Supplementary Figures

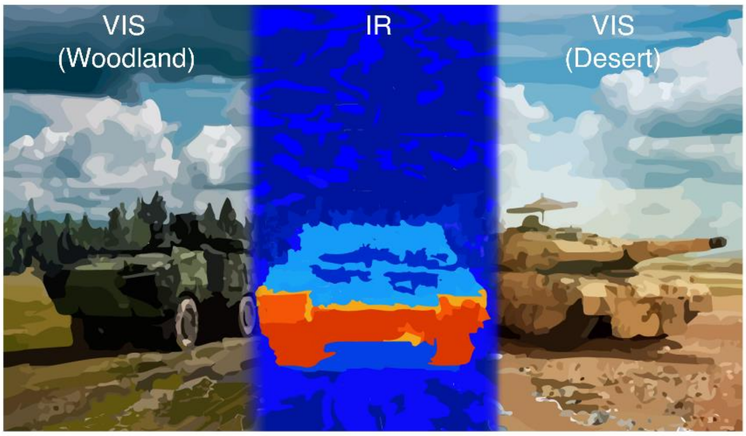

Supplementary Figure 1. Concept illustration of visible-infrared compatible camouflage in different landscapes. Reproduced with permission from Johan Jersblad. Copyright Saab AB.

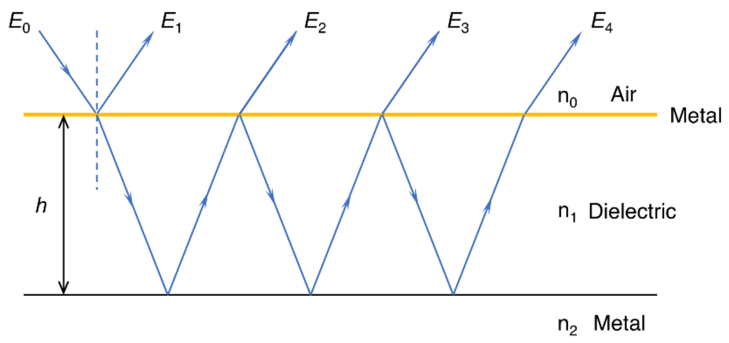

Supplementary Figure 2. Reflective and refractive behaviors of light with an angle of incidence in a three-layer system. The parameter n_0, n_1 and n_2 are refractive coefficients of corresponding materials. Detailed discussion is placed in Supplementary Note 1.

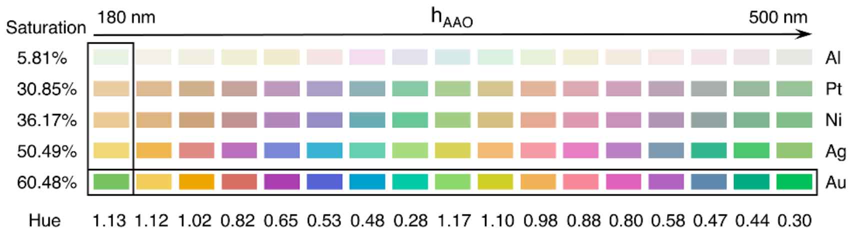

Supplementary Figure 3. Color representation of diverse materials for top layer and color saturation and hue depending on material and AAO layer thickness.

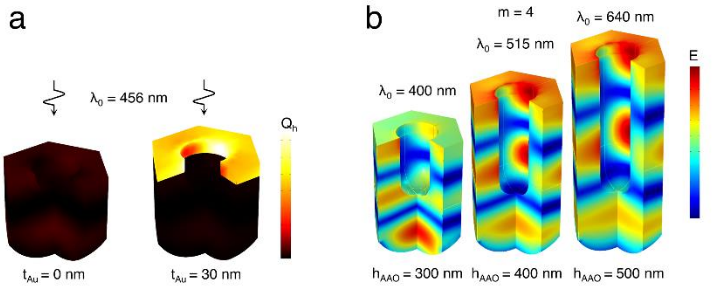

Supplementary Figure 4. a) Total power dissipation density distribution of the model in the presence and absence of Au layer. b) Local electric field distribution according to the thickness of the dielectric layer with same interference order (m = 4).

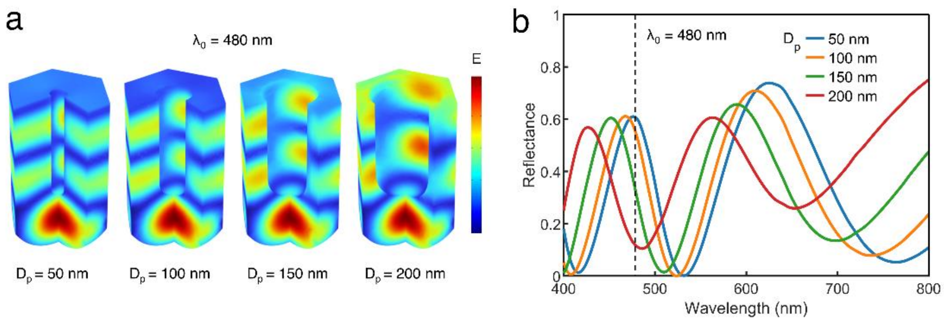

Supplementary Figure 5. a) Local electric field distribution according to the pore size of the AAO layer with same incident wavelength. b) Visible reflectance spectra of simulated results depending on the pore size of AAO layer.

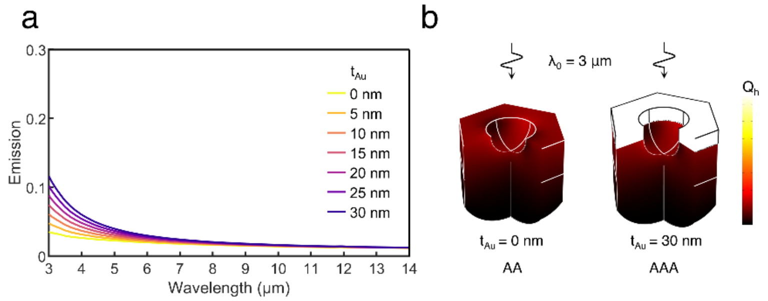

Supplementary Figure 6. a) Infrared emission spectra of simulated results depending on the Au thickness. b) Total power dissipation density distribution of the model in the presence and absence of Au layer.

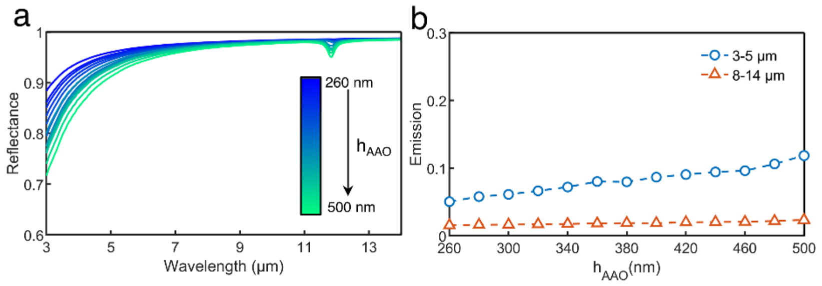

Supplementary Figure 7. a) Infrared reflectance spectra of simulated results depending on the AAO thickness. b) Infrared (MWIR and LWIR) emission of simulated results depending on the AAO thickness.

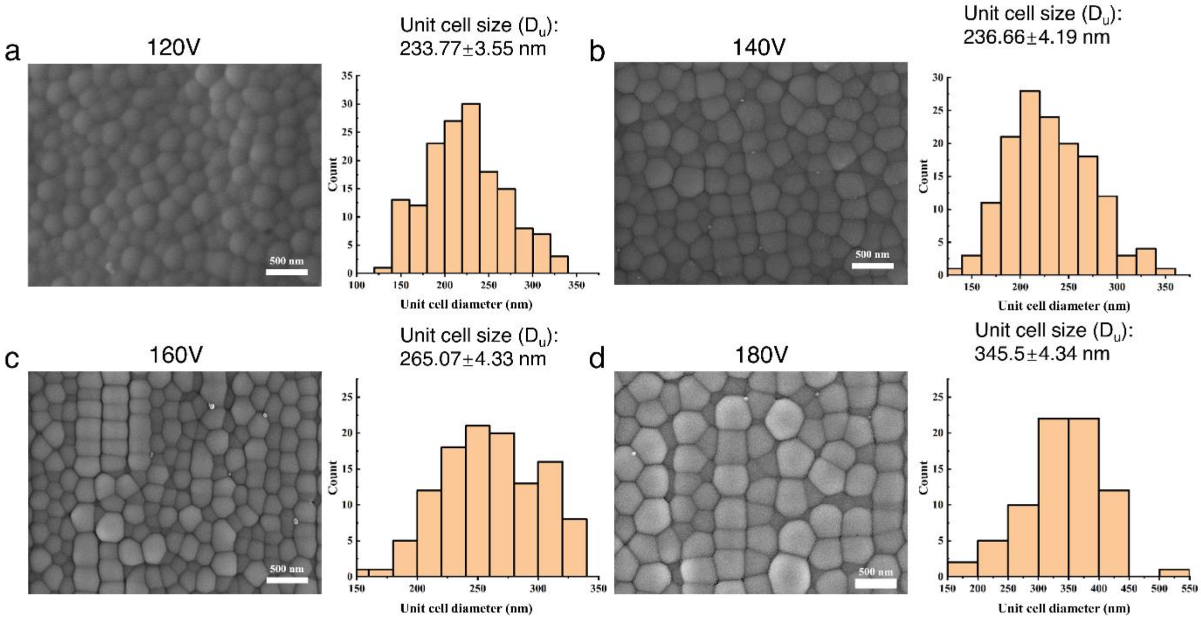

Supplementary Figure 8. The TEM images of the bottom of AAO layer and corresponding unit cell size of AAO under different voltages: a) 120 V; b) 140 V; c) 160 V; d) 180 V;

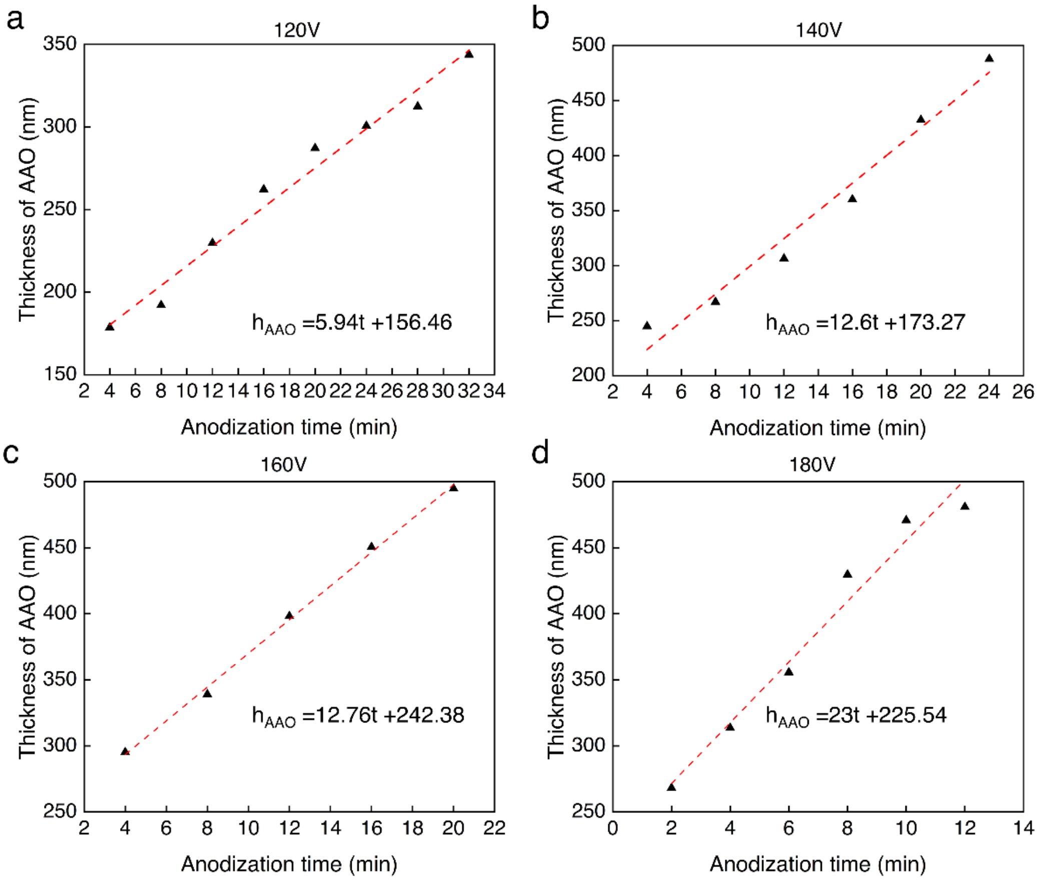

Supplementary Figure 9. The relationship between anodization time and the thickness of

AAO under different voltages: a) 120 V; b) 140 V; c) 160 V; d) 180 V;

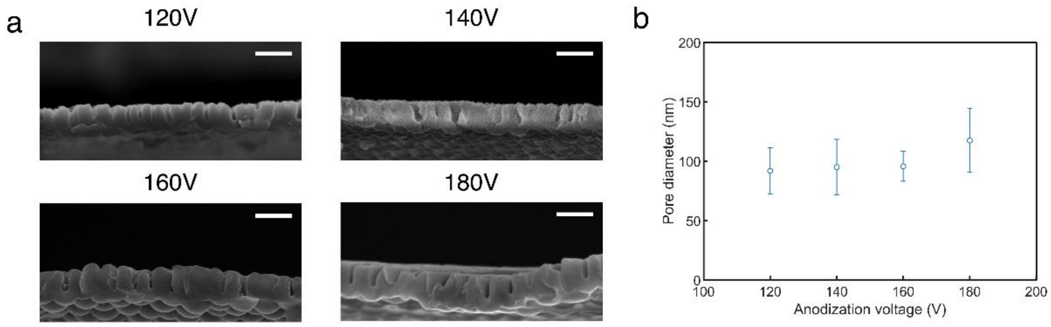

Supplementary Figure 10. Cross-sectional SEM images (a) and Measured pore diameters (b) of AAO under different voltages: 120 V-180 V. The scale bars of the SEM images are 500 nm.

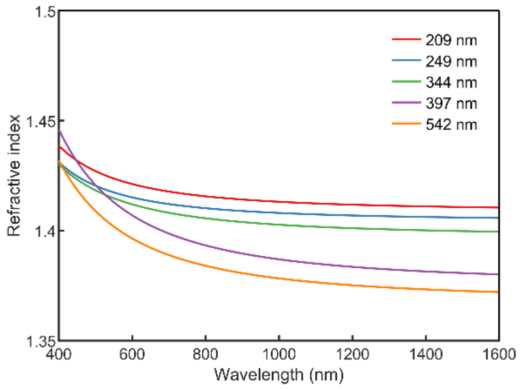

Supplementary Figure 11. Refractive index for different thicknesses of AAO under same oxidation voltage.

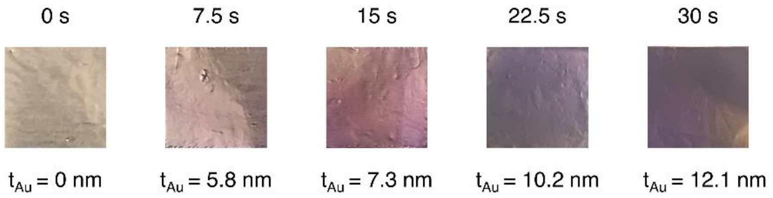

Supplementary Figure 12. Photographs of compatible camouflage films with different sputtering time and Au thickness.

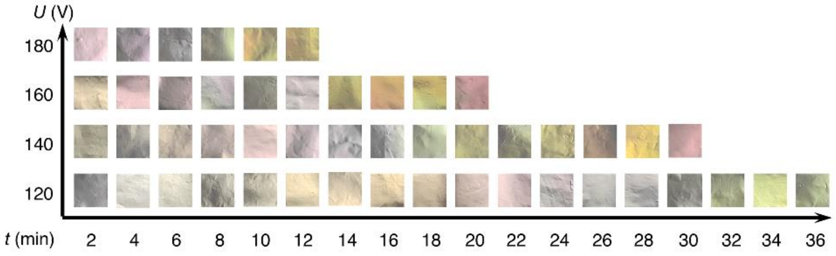

Supplementary Figure 13. Photographs of compatible camouflage films before top Au sputtering with different oxidation times and voltages.

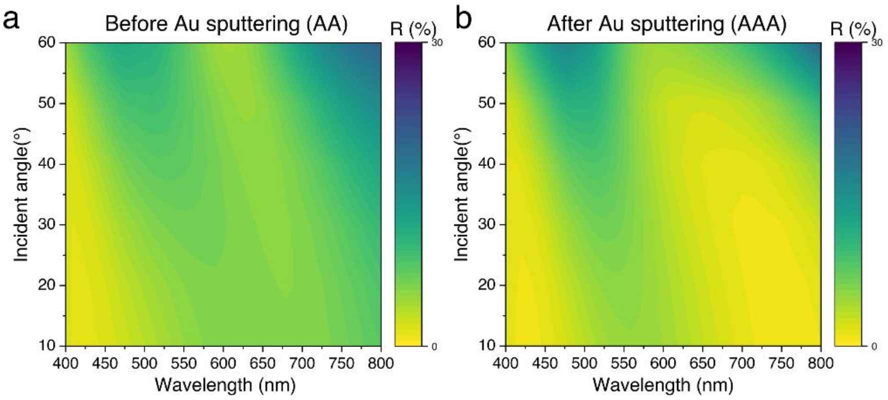

Supplementary Figure 14. Reflectance spectra of the compatible camouflage film with different incident angles in the visible range. a) Before top Au sputtering (AA); b) After top Au sputtering (AAA);

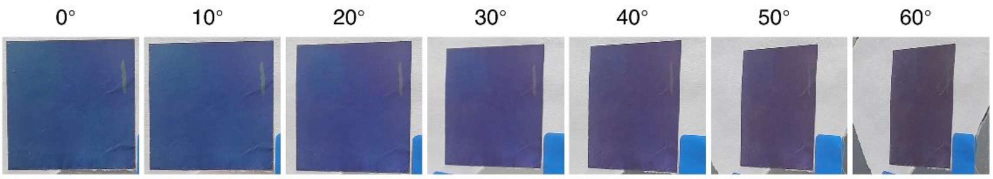

Supplementary Figure 15. The photographs taken at different viewing angles (0°- 60°) of camouflage films.

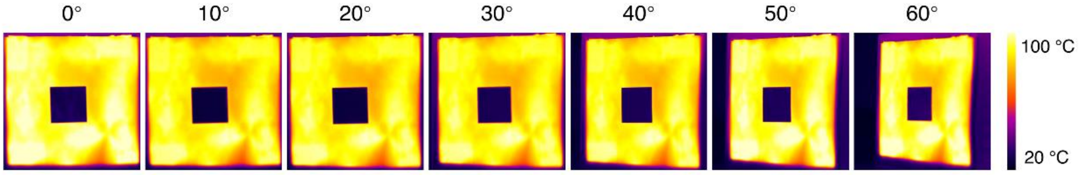

Supplementary Figure 16. The infrared images measured at different viewing angles (0°-60°) of camouflage films.

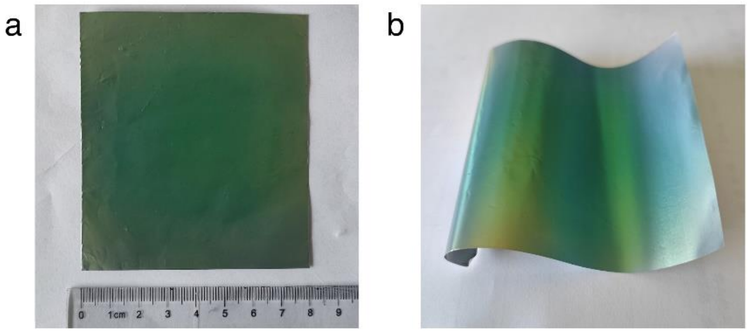

Supplementary Figure 17. a, b) Photograph of the flexible camouflage film.

Supplementary Note 1.

For a very thin metal layer, its thickness is close to zero. The structure can be simplified to be a typical three-layer structure. When a plane wave of monochromatic light is incident upon the interface between air and dielectric layer at angle θ. The reflective and refractive behaviors were described in the Supplementary Figure 2.

When the plane wave is normally incident to the structure. Let A^0 be the amplitude of the electric vector of the incident wave, which we assume to be linearly polarized. And For each member of the reflected or transmitted wave group, the difference in phase of the wave function from the previous member corresponds to twice the thickness of the dielectric layer. So, this phase difference is

$$\delta = \frac{4\pi}{\lambda_0} n_1 h_1 cos\theta, \#(1)$$

where h is the thickness of the dielectric layer and λ_0 is the wavelength in vacuum. Let r_{ab} be the reflection coefficient, and t_{ab} be the transmission coefficient for a wave travelling

from layer a to layer b. According to the Fresnel formulae, we have for a TE wave,

$$r_{ab} = \frac{n_a - n_b}{n_a + n_b}, \#(2)$$

$$t_{ab} = \frac{2n_a}{n_a + n_b}. \#(3)$$

And the relations between t and r is

$$tt' = T, \#(4)$$

$$r = -r', \#(5)$$

$$r^2 = r'^2 = R, \#(6)$$

where R and T, respectively the reflectivity and transmissivity of the structure surfaces, are related by

$$R + T = 1. \#(7)$$

The complex light filed of the waves reflected from surface are

$$E_1 = r_{01}A^0E_0e^{i\varphi_0},\ E_2 = r_{12}t_{10}t_{01}A^0E_0e^{i(\varphi_0+\delta)}, E_3 = r_{12}t_{10}t_{01}r_{10}r_{12}A^0E_0e^{i(\varphi_0+2\delta)}, \dots ,$$
$$E_p = r_{12}t_{10}t_{01}(r_{10}r_{12})^{p-2}A^0E_0e^{i[\varphi_0+(p-1)\delta]}, \dots \#(8)$$

If the first p reflected waves are superposed, the amplitude $A^r(p)$ of the electric vector of the reflected light is given by the expression

$$A^r(p) = [r_{01} + r_{12}t_{10}t_{01}e^{i\delta}(1 + r_{10}r_{12}e^{i\delta} + \cdots + (r_{10}r_{12})^{p-2}e^{i(p-2)\delta})]A^0$$
$$= \left[r_{01} + r_{12}t_{10}t_{01}e^{i\delta}\left(\frac{1-(r_{10}r_{12}e^{i\delta})^{p-2}}{1 - r_{10}r_{12}e^{i\delta}}\right)\right]A^0. \#(9)$$

If the surface of the structure is sufficiently large, the number of reflected waves is large; and in the limit as $p \to \infty$, we have from (9),

$$A^r \equiv A^r(\infty) = \frac{r_{01} + r_{12}(-r_{01}r_{10} + t_{10}t_{01})e^{i\delta}}{1 - r_{10}r_{12}e^{i\delta}}A^0. \#(10)$$

From (4), (5), (6) and (7) we can get

$$A^r = \frac{r_{01} + r_{12}e^{i\delta}}{1 - r_{10}r_{12}e^{i\delta}}A^0. \#(11)$$

So that the intensity $I^r = A^rA^{r*}$ of the reflected light is

$$I^r = \frac{r_{01}^2 + r_{12}^2 + 2r_{01}r_{12}\cos\delta}{1 + r_{01}^2 r_{12}^2 + 2\, r_{10}r_{12}\cos\delta} I^0, \#(12)$$

where $I^0 = A^0 A^{0*}$ is the intensity of the incident light.

For conventional F-P cavity, the reflection of top metal layer and bottom Al layer is very high, so $r_{01} \approx -r_{12} \to 1$. Then we put $r = r_{01}$, combined with relations (4)-(6), formula (11) can be rewritten as

$$I^r = \frac{(2 - 2\cos\delta\,)R}{1 + R^2 - 2R\cos\delta} I^0 = \frac{4R\sin^2\frac{\delta}{2}}{(1-R)^2 + 4R\sin^2\frac{\delta}{2}} I^0 = \frac{F\sin^2\frac{\delta}{2}}{1 + F\sin^2\frac{\delta}{2}} I^0, \#(13)$$

where the parameter F is defined by the formula

$$F = \frac{4R}{(1-R)^2}. \#(14)$$

The reflected intensity is minimal when

$$\sin^2\frac{\delta}{2} = 0, \delta = 2m\pi, \#(15)$$

where m is the order of interference and has integral value 1, 2, … So, there is a valley at $\delta = 2m\pi$ in the reflection spectrum. We use half-width, which is the width between two points on either side of a minimum where the intensity has increased to half its peak to valley difference, to assess how sharp the valley is. So, the reflection intensity is half its peak to valley difference when

$$\delta = 2m\pi \pm \frac{a}{2}, \#(16)$$

where by (13)

$$\frac{F\sin^2\frac{a}{4}}{1 + F\sin^2\frac{a}{4}} = 0 + \frac{1}{2}(1-0) = \frac{1}{2}. \#(17)$$

If R exceeds 0.9, F increases dramatically as R approaches 1, according to formula (14). When F is sufficiently large, a is so small that we may replace $sin(a/4)$ in formula (17) with a and get the half-width

$$a = \frac{4}{\sqrt{F}}. \#(18)$$

In conclusion, when the reflection coefficient of the surface is very high, F will be extremely large and the valley has a very small half-width, so the reflection spectrum exhibits

quite narrow valleys.

For semi-open F-P cavity, according to formula (12), the reflection of overall model is

$$R_{\text{model}} = \frac{I^r}{I^0} = \frac{r_{01}^2 + r_{12}^2 + 2r_{01}r_{12}\cos\delta}{1 + r_{01}^2 r_{12}^2 + 2\, r_{10}r_{12}\cos\delta}. \#(19)$$

If we set

$$H = n_2 h_2, \#(20)$$

We find from (19) that

$$\frac{\mathrm{d}R}{\mathrm{d}H} = 0, \sin\delta = 0, H = \frac{m\lambda_0}{4}, (m = 0, 1, 2, \dots). \#(21)$$

When m is odd, $H = \frac{\lambda_0}{4}, \frac{3\lambda_0}{4}, \frac{5\lambda_0}{4}, \dots$ then $\cos\delta = -1$ and $R_{\text{model}} = \left(\frac{r_{12}-r_{23}}{1-r_{12}r_{23}}\right)^2$.

When m is even, $H = \frac{2\lambda_0}{4}, \frac{4\lambda_0}{4}, \frac{6\lambda_0}{4}, \dots$ then $\cos\delta = 1$ and $R_{\text{model}} = \left(\frac{r_{12}+r_{23}}{1+r_{12}r_{23}}\right)^2$.

To determine whether the extreme value is maximum or minimum, we perform a second derivative calculation and find that when $H = \frac{m\lambda_0}{4}$ $(m = 1, 2, \dots)$

$$\frac{\mathrm{d}^2 R}{\mathrm{d}H^2} \gtreqless 0, (-1)^{\mathrm{m}} r_{12} r_{23} (1 + r_{12}^2\, r_{23}^2 - r_{12}^2 - r_{23}^2) \lesseqgtr 0. \#(22)$$

Then according to (2), we have

$$\begin{cases} \text{maximum, if } (-1)^{\mathrm{m}}(n_1 - n_2)(n_2 - n_3) > 0, \\ \text{minimum, if } (-1)^{\mathrm{m}}(n_1 - n_2)(n_2 - n_3) < 0. \end{cases} \#(23)$$
